# Supplementary material for: Proteomic Profiling of Endothelial Cell Secretomes After Exposure to Calciprotein Particles Reveals Downregulation of Basement Membrane Assembly and Increased Release of Soluble CD59
Source: Int J Mol Sci. 2024 Oct 23;25(21):11382. doi: 10.3390/ijms252111382 (PMC11546392; doi:10.3390/ijms252111382)

Figure S1. Volcano plots showing the differentially expressed proteins in primary human coronary artery endothelial cells (HCAEC) treated with Dulbecco's phosphate-buffered saline (DPBS), primary calciprotein particles (CPP-P), or secondary calciprotein particles (CPP-S) for 24 hours. Gray points depict the proteins with log_2fold change < 1 and FDR-corrected *p* value > 0.05, green points depict the proteins with log_2fold change > 1 and FDR-corrected *p* value > 0.05, blue points depict the proteins with log_2fold change < 1 and FDR-corrected *p* value < 0.05, and red points depict the proteins with log_2fold change > 1 and FDR-corrected *p* value < 0.05 (termed as differentially expressed proteins).

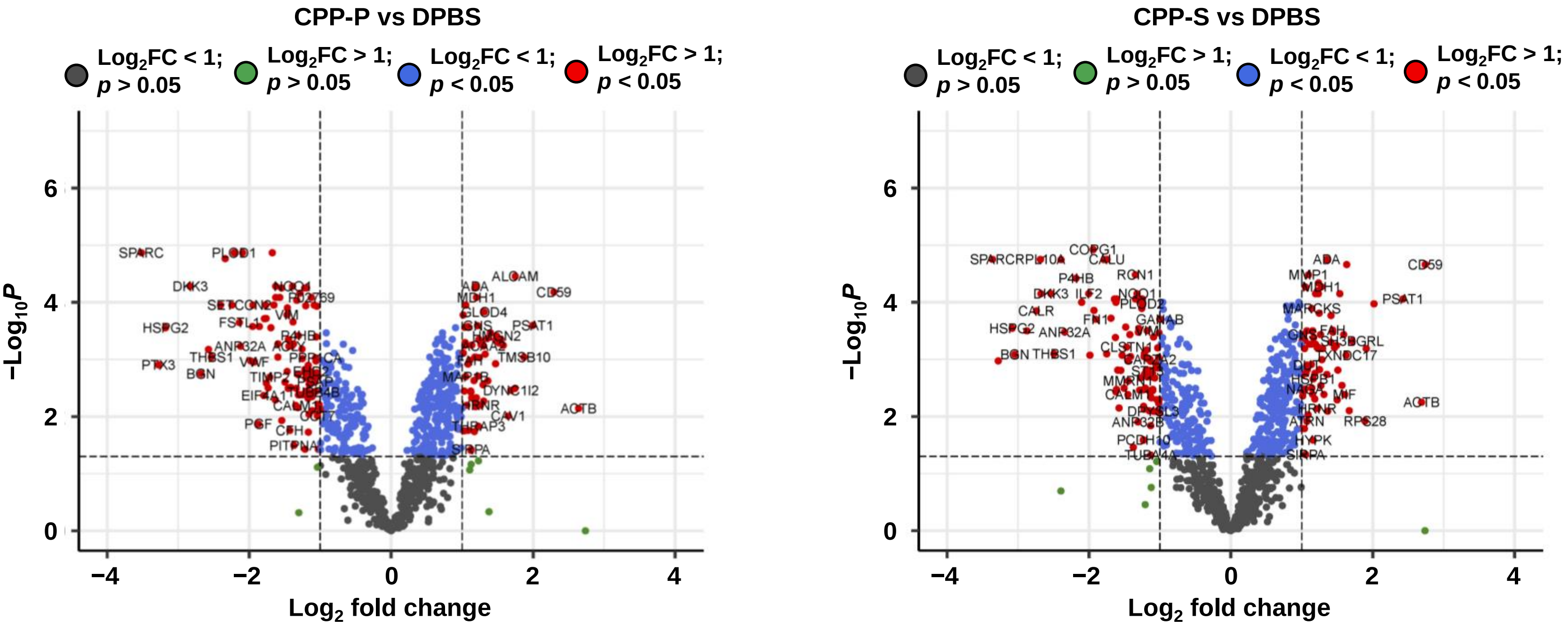

Figure S2. Volcano plots showing the differentially expressed proteins in primary human coronary artery endothelial cells (HCAEC) treated with magnesiprotein particles (MPPs), primary calciprotein particles (CPP-P), or secondary calciprotein particles (CPP-S) for 24 hours. Gray points depict the proteins with $\log_2$fold change < 1 and FDR-corrected *p* value > 0.05, green points depict the proteins with $\log_2$fold change > 1 and FDR-corrected *p* value > 0.05, blue points depict the proteins with $\log_2$fold change < 1 and FDR-corrected *p* value < 0.05, and red points depict the proteins with $\log_2$fold change > 1 and FDR-corrected *p* value < 0.05 (termed as differentially expressed proteins).

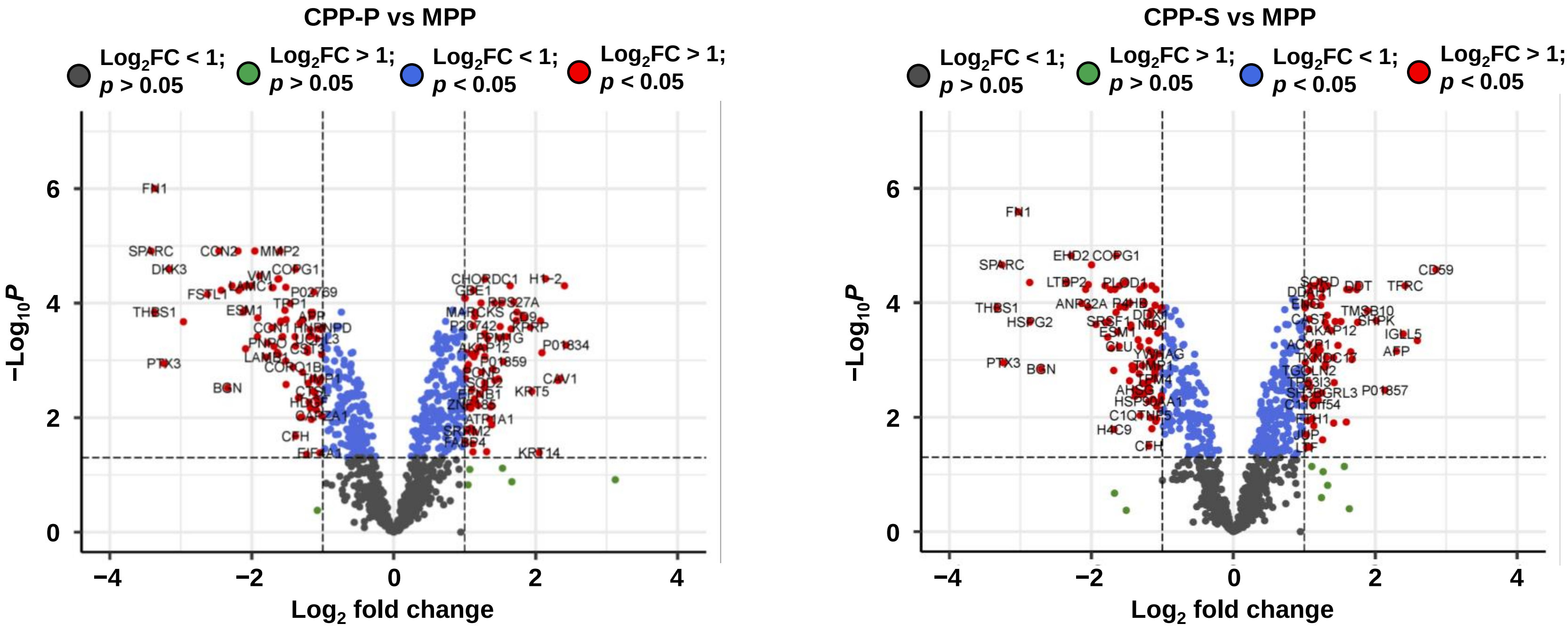

Figure S3. Volcano plots showing the differentially expressed proteins in primary human internal thoracic artery endothelial cells (HITAEC) treated with Dulbecco's phosphate-buffered saline (DPBS), primary calciprotein particles (CPP-P), or secondary calciprotein particles (CPP-S) for 24 hours. Gray points depict the proteins with $\log_2$fold change < 1 and FDR-corrected *p* value > 0.05, green points depict the proteins with $\log_2$fold change > 1 and FDR-corrected *p* value > 0.05, blue points depict the proteins with $\log_2$fold change < 1 and FDR-corrected *p* value < 0.05, and red points depict the proteins with $\log_2$fold change > 1 and FDR-corrected *p* value < 0.05 (termed as differentially expressed proteins).

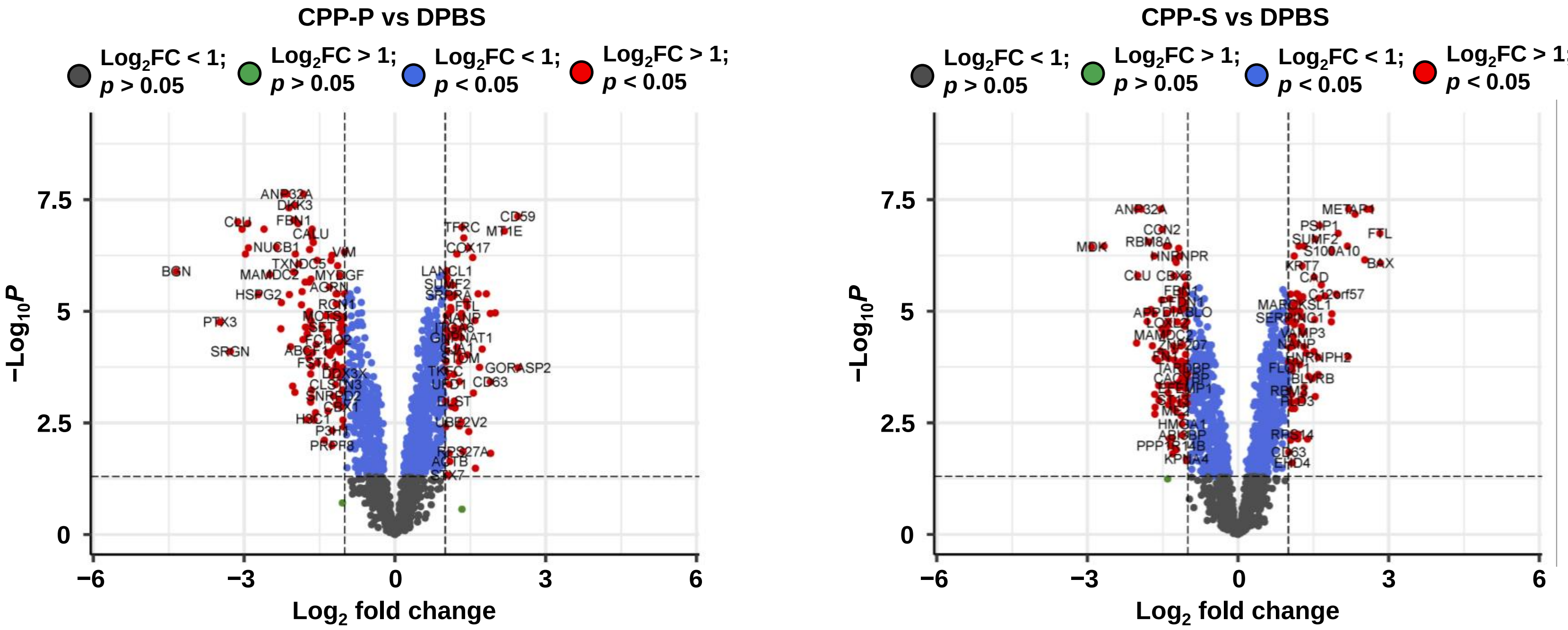

Figure S4. Volcano plots showing the differentially expressed proteins in primary human internal thoracic artery endothelial cells (HITAEC) treated with magnesiprotein particles (MPPs), primary calciprotein particles (CPP-P), or secondary calciprotein particles (CPP-S) for 24 hours. Gray points depict the proteins with $\log_2$fold change < 1 and FDR-corrected *p* value > 0.05, green points depict the proteins with $\log_2$fold change > 1 and FDR-corrected *p* value > 0.05, blue points depict the proteins with $\log_2$fold change < 1 and FDR-corrected *p* value < 0.05, and red points depict the proteins with $\log_2$fold change > 1 and FDR-corrected *p* value < 0.05 (termed as differentially expressed proteins).

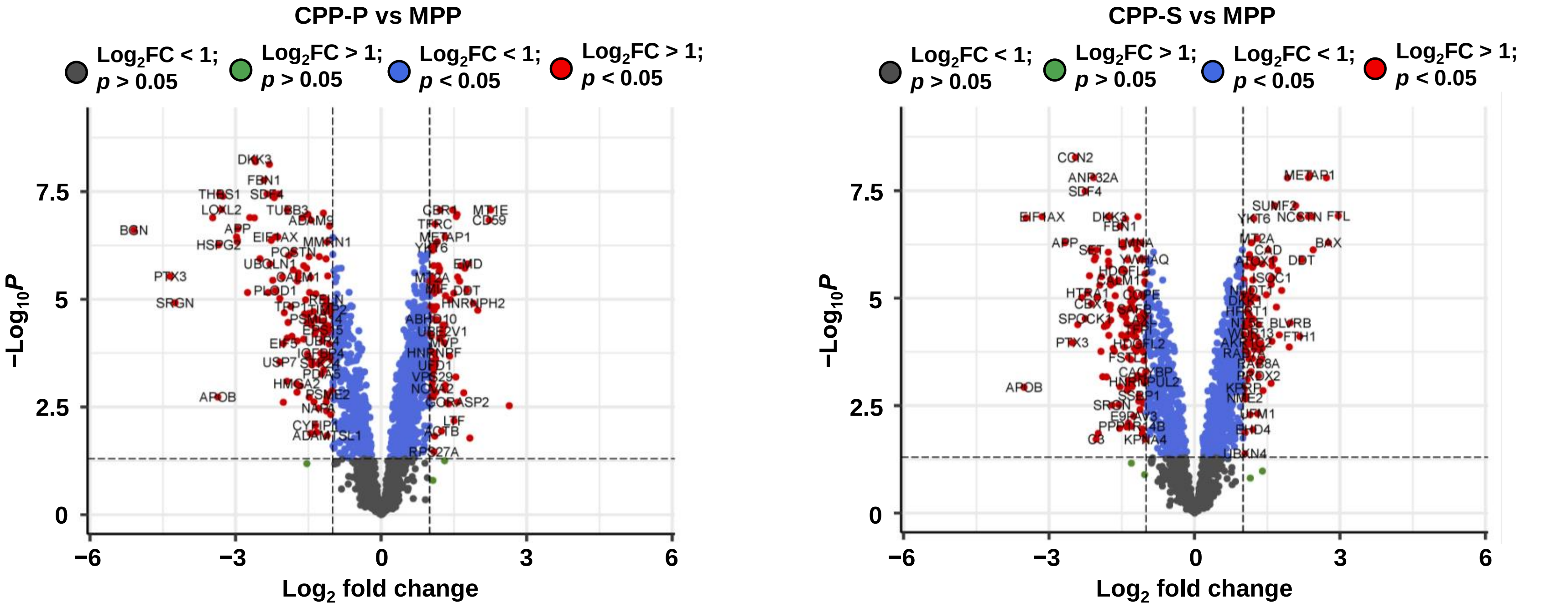

Figure S5. Volcano plots showing the differentially expressed proteins in primary human coronary artery endothelial cells (HCAEC) and primary human internal thoracic artery endothelial cells (HITAEC) treated with Dulbecco's phosphate-buffered saline (DPBS) or magnesiprotein particles (MPPs) for 24 hours. Gray points depict the proteins with $\log_2$fold change < 1 and FDR-corrected *p* value > 0.05, green points depict the proteins with $\log_2$fold change > 1 and FDR-corrected *p* value > 0.05, blue points depict the proteins with $\log_2$fold change < 1 and FDR-corrected *p* value < 0.05, and red points depict the proteins with $\log_2$fold change > 1 and FDR-corrected *p* value < 0.05 (termed as differentially expressed proteins).

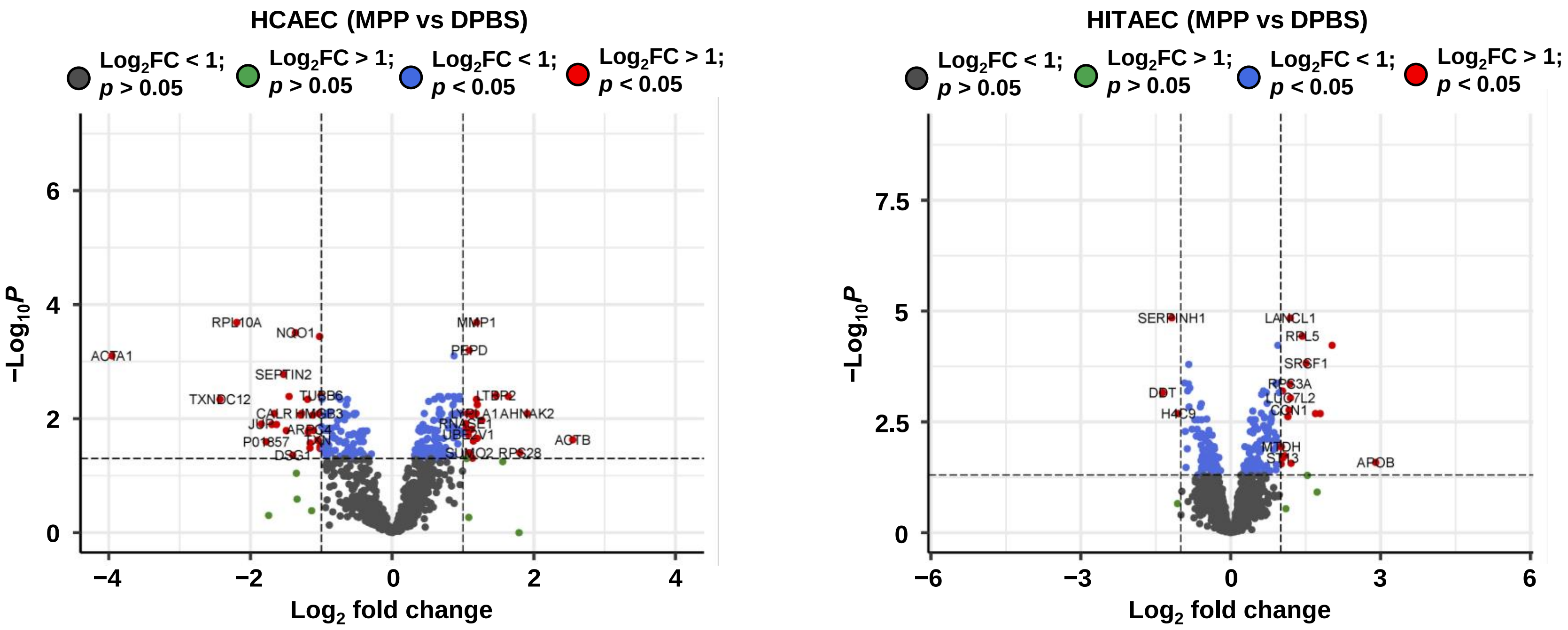

Figure S6. Volcano plots showing the differentially expressed proteins in primary human coronary artery endothelial cells (HCAEC) and primary human internal thoracic artery endothelial cells (HITAEC) treated with primary calciprotein particles (CPP-P) or secondary calciprotein particles (CPP-S) for 24 hours. Gray points depict the proteins with $\log_2$fold change < 1 and FDR-corrected *p* value > 0.05, green points depict the proteins with $\log_2$fold change > 1 and FDR-corrected *p* value > 0.05, blue points depict the proteins with $\log_2$fold change < 1 and FDR-corrected *p* value < 0.05, and red points depict the proteins with $\log_2$fold change > 1 and FDR-corrected *p* value < 0.05 (termed as differentially expressed proteins).

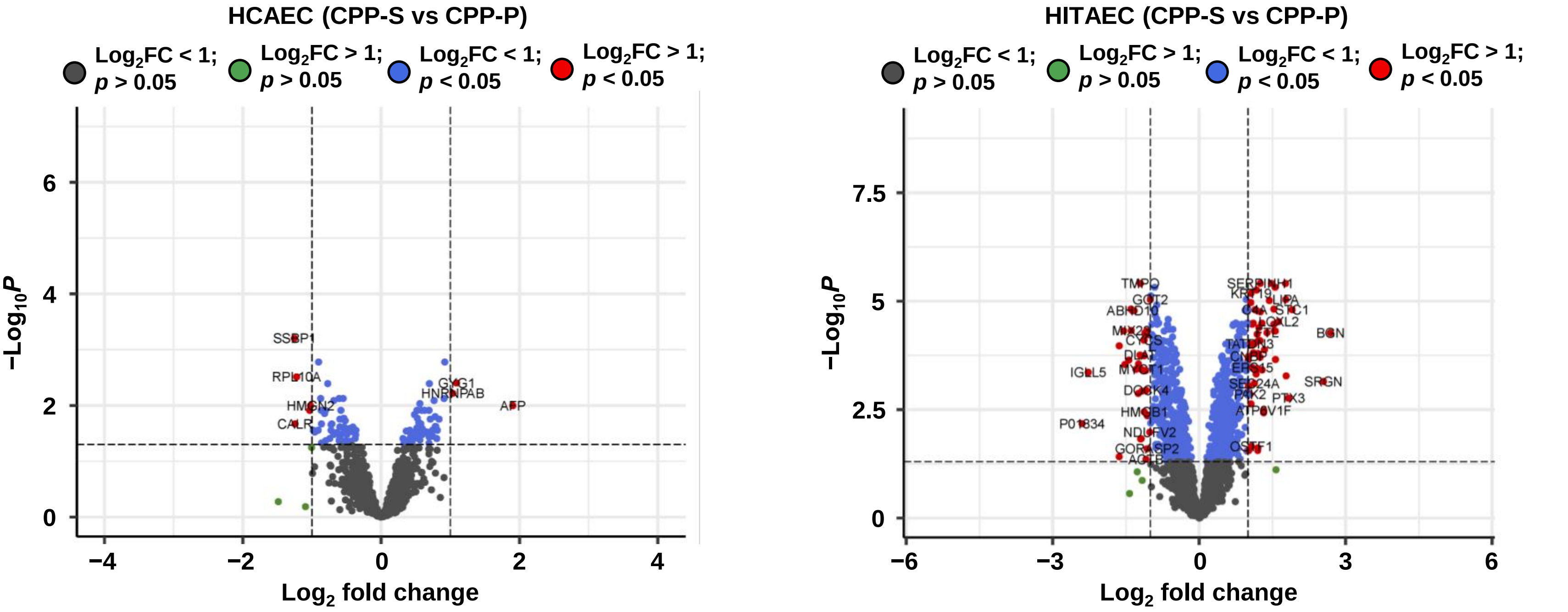

Figure S7. Volcano plots showing the differentially expressed proteins in Dulbecco's phosphate-buffered saline (DPBS)-treated and magnesiprotein particle (MPP)-treated primary human coronary artery endothelial cells (HCAEC) and primary human internal thoracic artery endothelial cells (HITAEC). DPBS and MPP groups have been pooled for the statistical significance purposes. Gray points depict the proteins with $\log_2$fold change < 1 and FDR-corrected *p* value > 0.05, green points depict the proteins with $\log_2$fold change > 1 and FDR-corrected *p* value > 0.05, blue points depict the proteins with $\log_2$fold change < 1 and FDR-corrected *p* value < 0.05, and red points depict the proteins with $\log_2$fold change > 1 and FDR-corrected *p* value < 0.05 (termed as differentially expressed proteins).

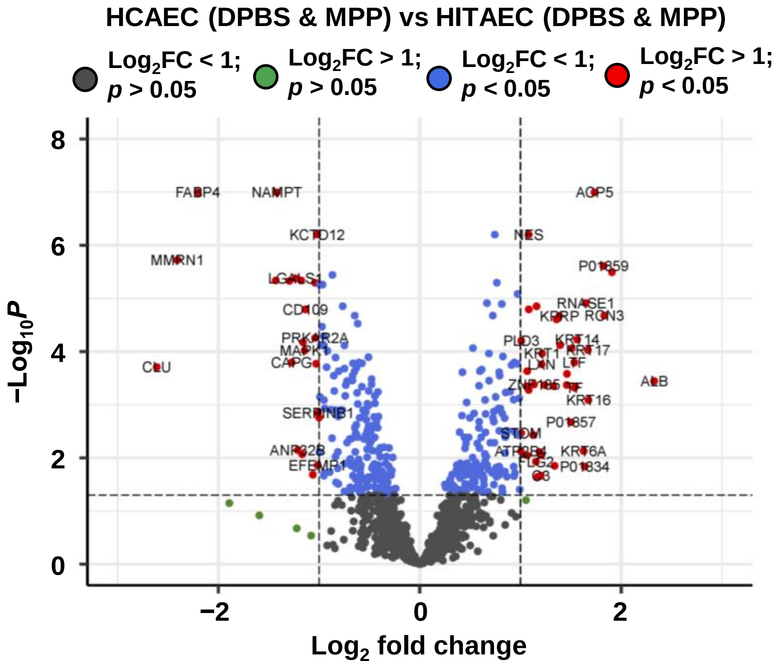

Figure S8. Analysis of protein-protein interactions among the upregulated proteins in secretomes from primary human coronary artery endothelial cells (HCAEC) treated with Dulbecco's phosphate-buffered saline (DPBS), magnesiprotein particles (MPP), primary calciprotein particles (CPP-P), or secondary calciprotein particles (CPP-S) for 24 hours. (a) CPP-P vs DPBS; (b) CPP-P vs MPP; (c) CPP-S vs DPBS; (d) CPP-S vs MPP. Analysis was performed through the Search Tool for the Retrieval of Interacting Genes/Proteins (STRING).

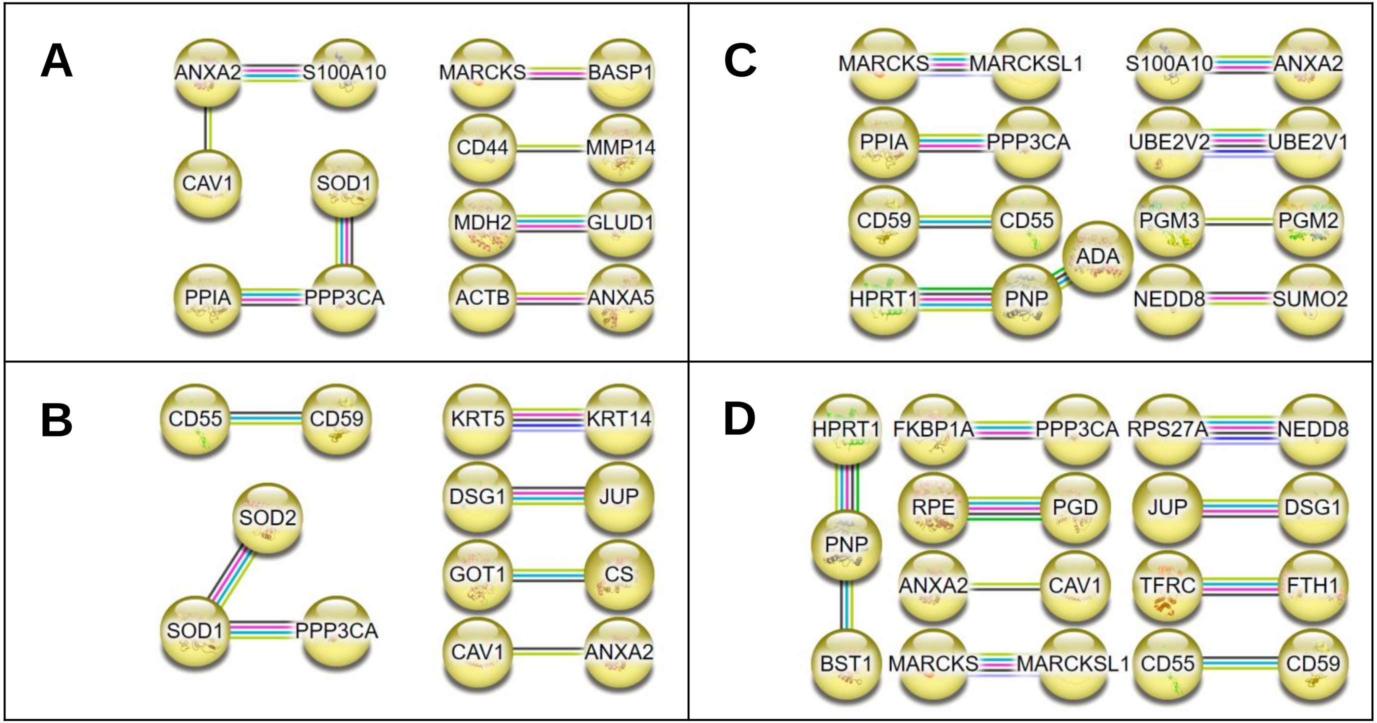

Figure S9. Analysis of protein-protein interactions among the upregulated proteins in secretomes from primary human internal thoracic artery endothelial cells (HITAEC) treated with Dulbecco's phosphate-buffered saline (DPBS), magnesiprotein particles (MPP), primary calciprotein particles (CPP-P), or secondary calciprotein particles (CPP-S) for 24 hours. (a) CPP-P vs DPBS; (b) CPP-P vs MPP; (c) CPP-S vs DPBS; (d) CPP-S vs MPP. Analysis was performed through the Search Tool for the Retrieval of Interacting Genes/Proteins (STRING).

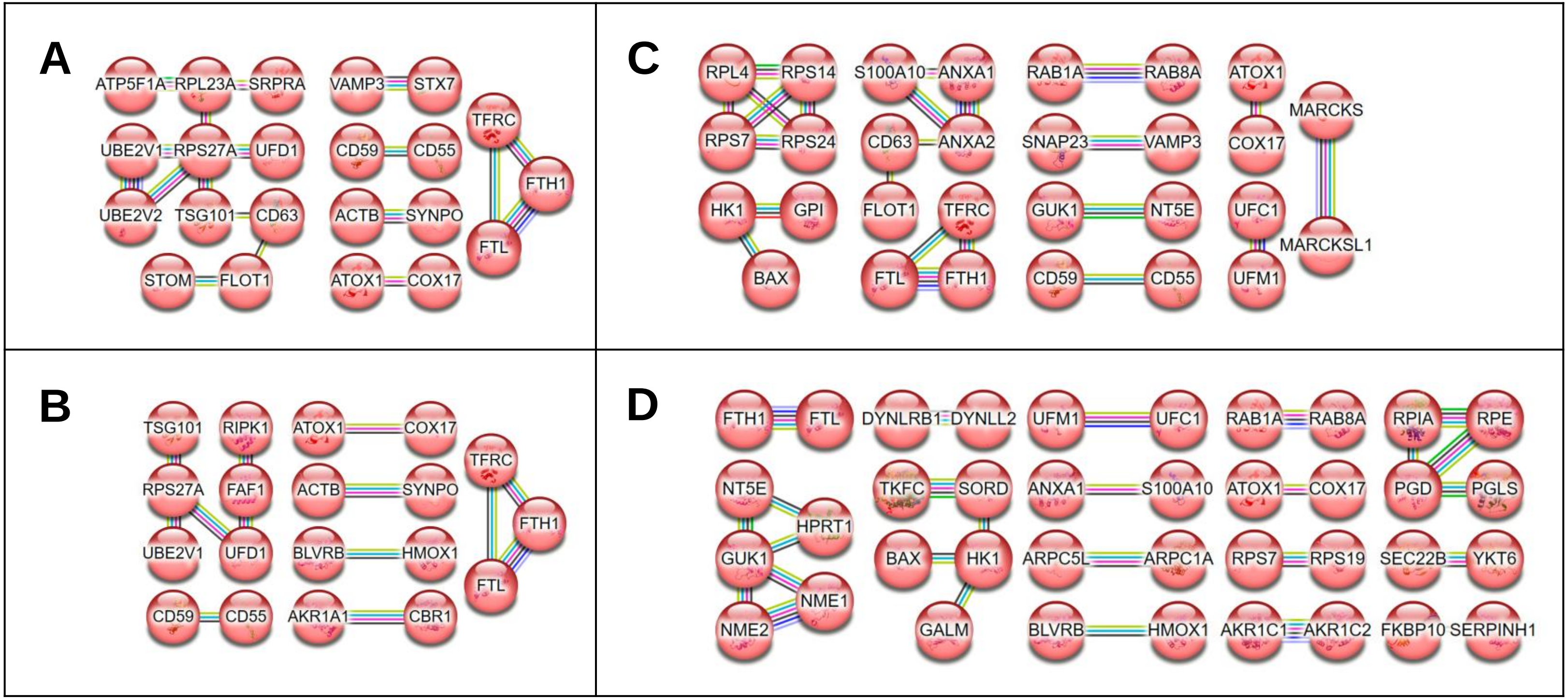

Figure S10. Analysis of protein-protein interactions among the upregulated and downregulated proteins in secretomes from primary human coronary artery endothelial cells (HCAEC) and primary human internal thoracic artery endothelial cells (HITAEC) treated with magnesiprotein particles (MPP) or Dulbecco's phosphate-buffered saline (DPBS) for 24 hours. (a) HCAEC, MPP vs. DPBS, upregulated proteins; (b) HCAEC, MPP vs. DPBS, downregulated proteins; (c) HITAEC, MPP vs. DPBS, upregulated proteins; (d) HITAEC, MPP vs. DPBS, downregulated proteins. Analysis was performed through the Search Tool for the Retrieval of Interacting Genes/Proteins (STRING).

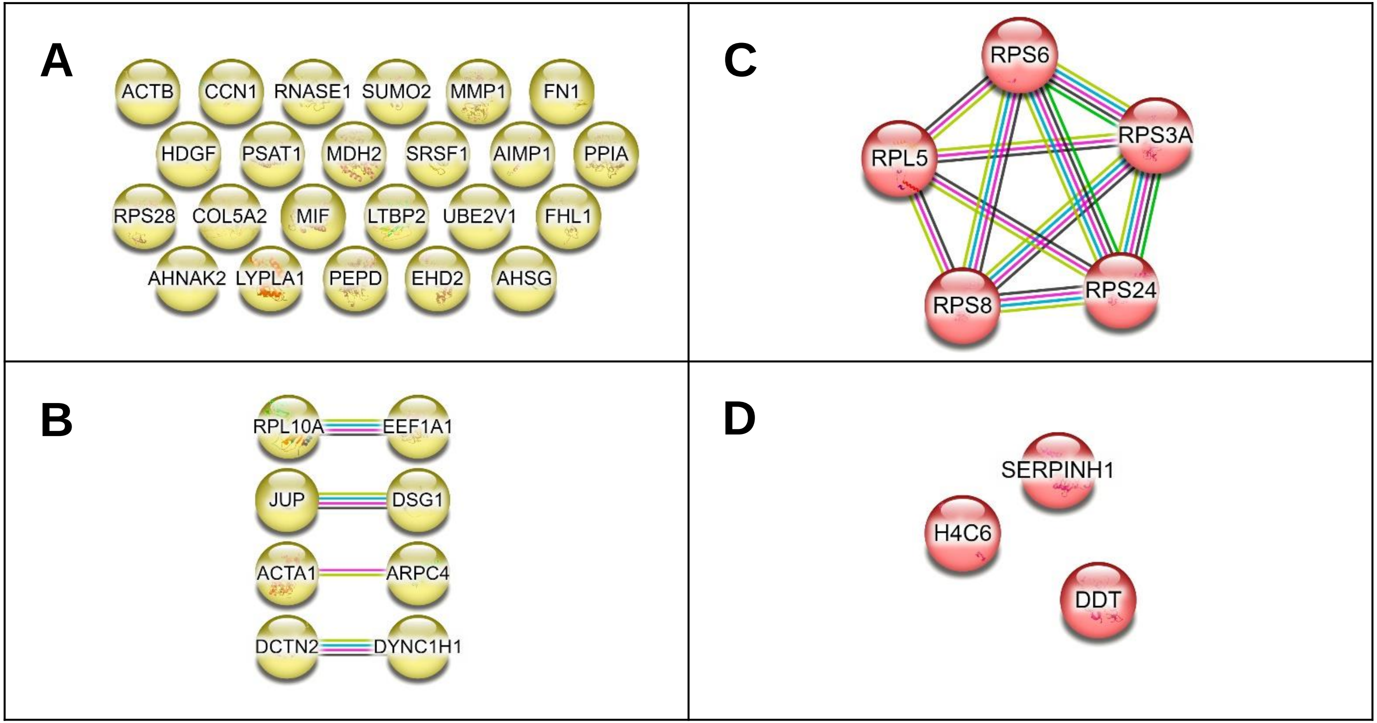

Figure S11. Analysis of protein-protein interactions among the upregulated and downregulated proteins in secretomes from primary human coronary artery endothelial cells (HCAEC) and primary human internal thoracic artery endothelial cells (HITAEC) treated with primary calciprotein particles (CPP-P) or secondary calciprotein particles (CPP-S) for 24 hours. (a) HCAEC, CPP-S vs. CPP-P, upregulated proteins; (b) HCAEC, CPP-S vs. CPP-P, downregulated proteins; (c) HITAEC, CPP-S vs. CPP-P, upregulated proteins; (d) HITAEC, CPP-S vs. CPP-P, downregulated proteins. Analysis was performed through the Search Tool for the Retrieval of Interacting Genes/Proteins (STRING).

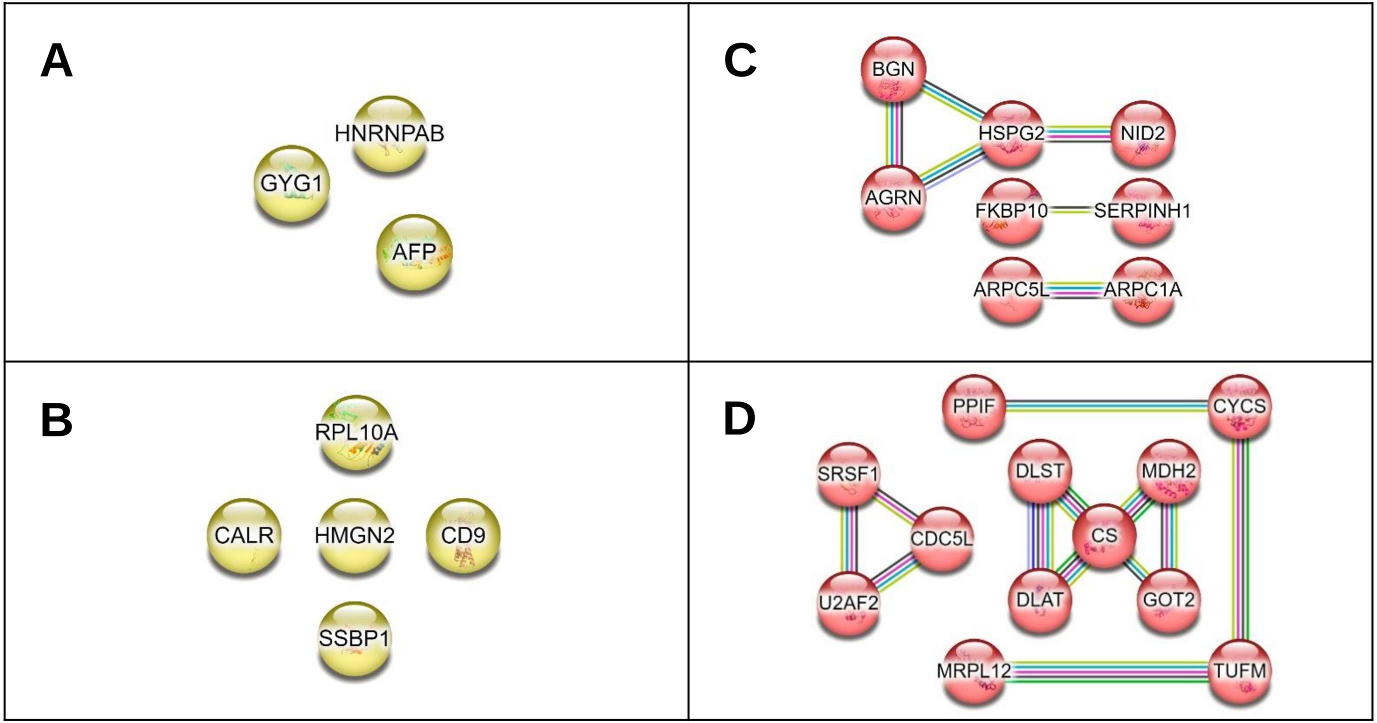

Figure S12. Analysis of protein-protein interactions among the proteins upregulated in the secretome from primary human coronary artery endothelial cells (HCAEC) as compared to the secretome from primary human internal thoracic artery endothelial cells (HITAEC) treated with Dulbecco's phosphate-buffered saline (DPBS) or magnesiprotein particles (MPP). DPBS and MPP groups were pooled to increase the sample size. Analysis was performed through the Search Tool for the Retrieval of Interacting Genes/Proteins (STRING).

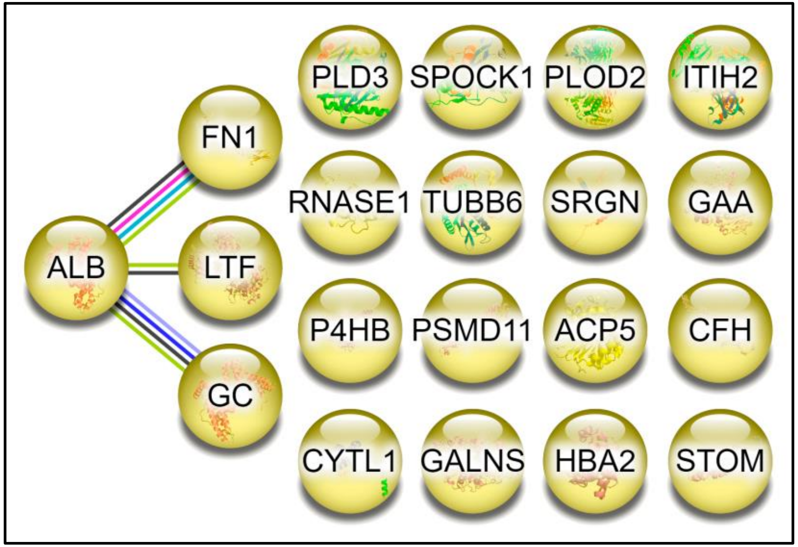

Figure S13. Analysis of protein-protein interactions among the proteins upregulated in the secretome from primary human internal thoracic artery endothelial cells (HITAEC) as compared to the secretome from primary human coronary artery endothelial cells (HCAEC) treated with Dulbecco's phosphate-buffered saline (DPBS) or magnesiprotein particles (MPP). DPBS and MPP groups were pooled to increase the sample size. Analysis was performed through the Search Tool for the Retrieval of Interacting Genes/Proteins (STRING).

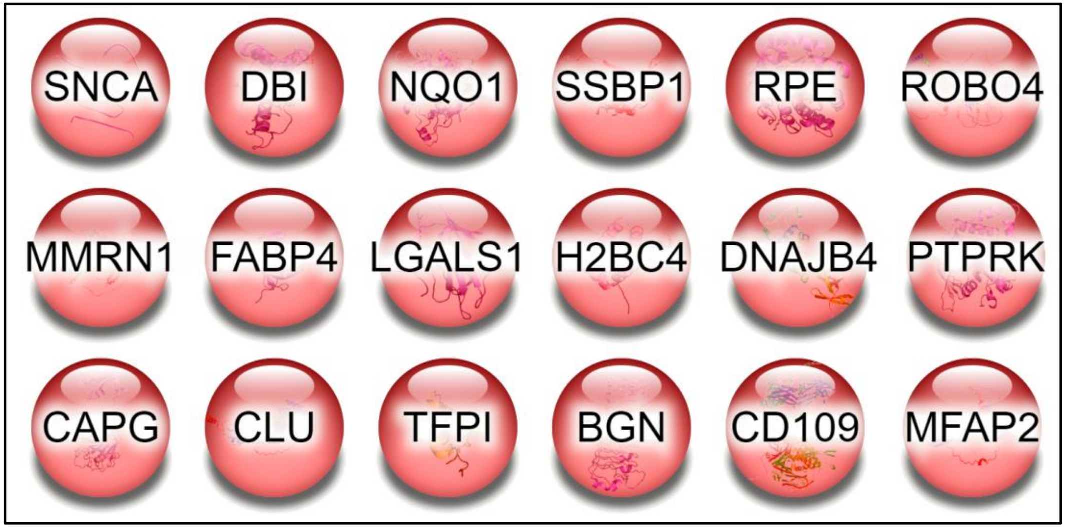

Supplement: Supplementary file 1 [file ijms-25-11382-s001.zip › Supplementary Figures (S1-S13).pdf]
